# Aggregated transthyretin is specifically packaged into placental nano-vesicles in preeclampsia

**DOI:** 10.1038/s41598-017-07017-x

**Published:** 2017-07-27

**Authors:** Mancy Tong, Shi-bin Cheng, Qi Chen, Joana DeSousa, Peter R. Stone, Joanna L. James, Lawrence W. Chamley, Surendra Sharma

**Affiliations:** 1 Department of Obstetrics and Gynaecology, The University of Auckland, Auckland, 1142 New Zealand; 2 Department of Pediatrics, Women and Infants Hospital-Warren Alpert Medical School of Brown University, Providence, Rhode Island USA; 3 Maternal Fetal Medicine, Auckland City Hospital, Auckland, New Zealand

## Abstract

In preeclampsia, the serum levels of transthyretin, a carrier protein for thyroxine, are elevated. Transthyretin isolated from preeclamptic serum is also aggregated and can induce preeclampsia-like symptoms in pregnant IL10^−/−^ mice. Using western blotting, immunofluorescence, ELISA and qRT-PCR, we investigated the production of transthyretin by preeclamptic placentae and whether transthyretin is carried into the maternal circulation via placental extracellular vesicles. Both total and aggregated transthyretin were present in higher levels in preeclamptic placentae compared to normotensive placentae (p < 0.05, n = 7), however the levels of transythretin mRNA were not significantly different (n = 8). Preeclamptic placentae secreted similar levels of total transthyretin compared to normotensive placentae (2352 ± 2949 ng/mL vs. 3250 ± 1864 ng/mL, mean ± SD, p > 0.05, n = 8), however in preeclampsia, a significant proportion is vesicle-associated (~48% vs 0%). Increased levels of aggregated transthyretin were specifically associated to preeclamptic nano-vesicles (p < 0.02, n = 8). This study showed that the placenta actively produces transthyretin and in preeclampsia, a significant amount is extruded into the maternal circulation via placental exracellular vesicles. The increased aggregation of transthyretin in preeclampsia occurs at the post-transcriptional level and while preeclamptic nano-vesicles may be removing a toxic aggregated protein from the placenta, they may also be delivering aggregated transthyretin to specific maternal organs, contributing to the pathogenesis of preeclampsia.

## Introduction

Preeclampsia is a hypertensive disease of pregnancy which affects 5–8% of otherwise healthy pregnant women^1–3^. This disease is characterized by *de novo* hypertension and proteinuria after 20 weeks of gestation^1^. While the pathogenesis of preeclampsia remains unclear, the placenta is known to play a crucial role as this disease occurs only in pregnancy or in patients with placental tumours; and symptoms are usually quickly alleviated by the delivery of the placenta^4, 5^. It is currently hypothesized that altered blood flow to the placenta during its development causes the release of placental toxins into the maternal circulation which trigger the clinical symptoms of preeclampsia^6^.

The nature of the placental toxins that trigger preeclampsia are not known, but extracellular vesicles (lipid-enclosed packages of proteins and nucleic acids) are being increasingly recognised as important mediators of feto-maternal communication. It has been hypothesized that, in preeclampsia, these vesicles may be, or carry, placental toxins^7–9^. All cells produce extracellular vesicles but the syncytiotrophoblast, a multinucleated cell that covers the entire surface of the human placenta, produces an unusually large range of extracellular vesicles, ranging from multinucleated syncytial nuclear aggregates (macro-vesicles), to subcellular micro-vesicles and nano-vesicles (a component of which are exosomes)^10^. As the human syncytiotrophoblast is bathed in maternal blood throughout most of gestation, placental extracellular vesicles that are extruded by the syncytiotrophoblast can enter the maternal circulation via the uterine veins and interact with different maternal organs and target cells.

Transthyretin is a 54 kDa homotetrameric protein transporter of thyroxine and retinol binding protein^11^. Transthyretin is mainly synthesised in the liver and choroid plexus, but can also be produced by the placenta^12, 13^. The production of transthyretin by the placenta is crucial for fetal development in the first trimester as the fetus is not able to produce its own thyroid hormone until 16 weeks of gestation and must rely on maternally supplied thyroxine carried by transthyretin for neural development^14, 15^.

The levels of transthyretin have been shown to be altered in several amyloid diseases, such as familial amyloid cardiomyopathy, polyneuropathy and senile systemic amyloidosis, where transthyretin is deposited onto tissues as toxic aggregates^11, 16^. Recently, increased levels of transthyretin have also been reported on placental tissue and in serum of women with preeclampsia^17^. In addition, the administration of transthyretin from preeclamptic serum into pregnant IL10^−/−^ mice induced the hallmark symptoms of preeclampsia (hypertension, proteinuria, glomerular endotheliosis, fetal growth restriction [FGR]) whereas, transthyretin from control serum did not^17^. These lines of evidence suggest that transthyretin may be involved in the early pathogenesis of preeclampsia, however the source of pathological transthyretin remains unclear. Therefore, this study was undertaken to investigate whether human placentae produce altered levels of transthyretin in preeclampsia and whether transthyretin is carried from the placenta into the maternal circulation via extracellular vesicles.

## Results

### The levels of transthyretin protein but not mRNA were increased in preeclamptic compared to normotensive placentae

**Figure 1:**
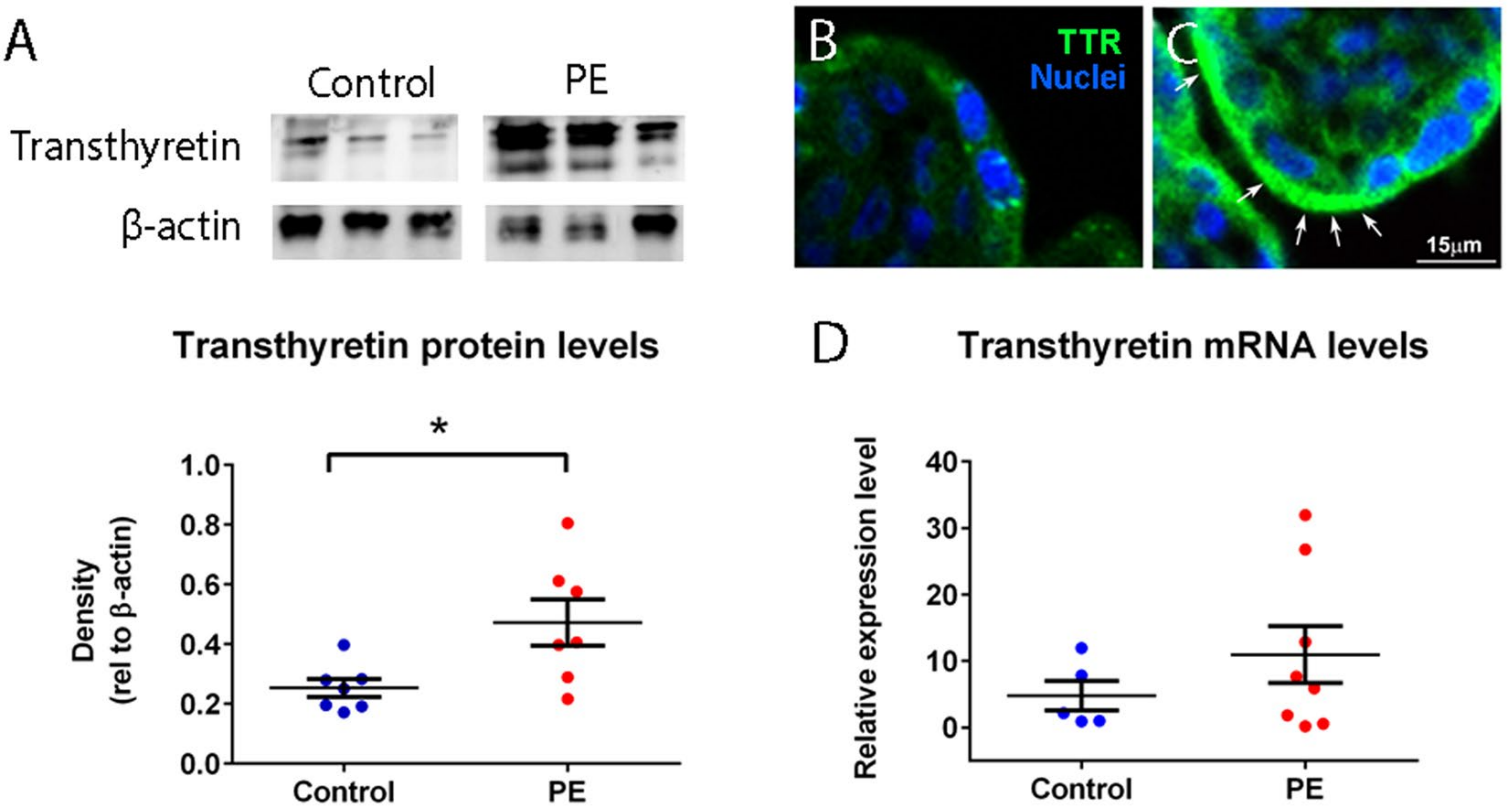
Transthyretin expression by preeclamptic placentae. Western blotting showed significantly higher levels of transthyretin in preeclamptic placentae compared to control placentae (*p = 0.0212, n = 7, **A**). Full-length blots are presented in Supplementary Figure 1. Immunofluorescent staining for transthyretin on placental sections from normotensive term (**B**) and preeclamptic pregnancies (**C**) showed higher levels of transthyretin in preeclamptic placentae. The granular staining of transthyretin are arrowed and can be seen most clearly in the control. Quantitative RT-PCR suggests that the mRNA levels of transthyretin were not different between preeclamptic and control placental explants (n = 8, **D**). Mean and SEM are depicted.

As it has previously been reported that transthyretin is present at higher levels in preeclamptic placentae compared to normotensive placentae, and transthyretin staining is colocalised with thioflavin S staining, suggesting aggregation^17^, in this study, we further investigated the production of transthyretin by preeclamptic placentae. Semi-quantitative western blotting under non-reducing conditions showed significantly increased levels of aggregated transthyretin in preeclamptic placentae compared to control normotensive placentae (p = 0.0212, n = 7, Fig. 1A). Interestingly, while there was clearly more transthyretin protein present in cytoplasmic granules within the synytiotrophoblast of preeclamptic placentae compared to that of normotensive placentae (Fig. 1B–C), the levels of transthyretin mRNA transcripts were not significantly different between preeclamptic (n = 8) and normotensive (n = 5) placentae (p > 0.05, Fig. 1D).

### Transthyretin is associated with extracellular vesicles from both first trimester and term human placentae

**Figure 2:**
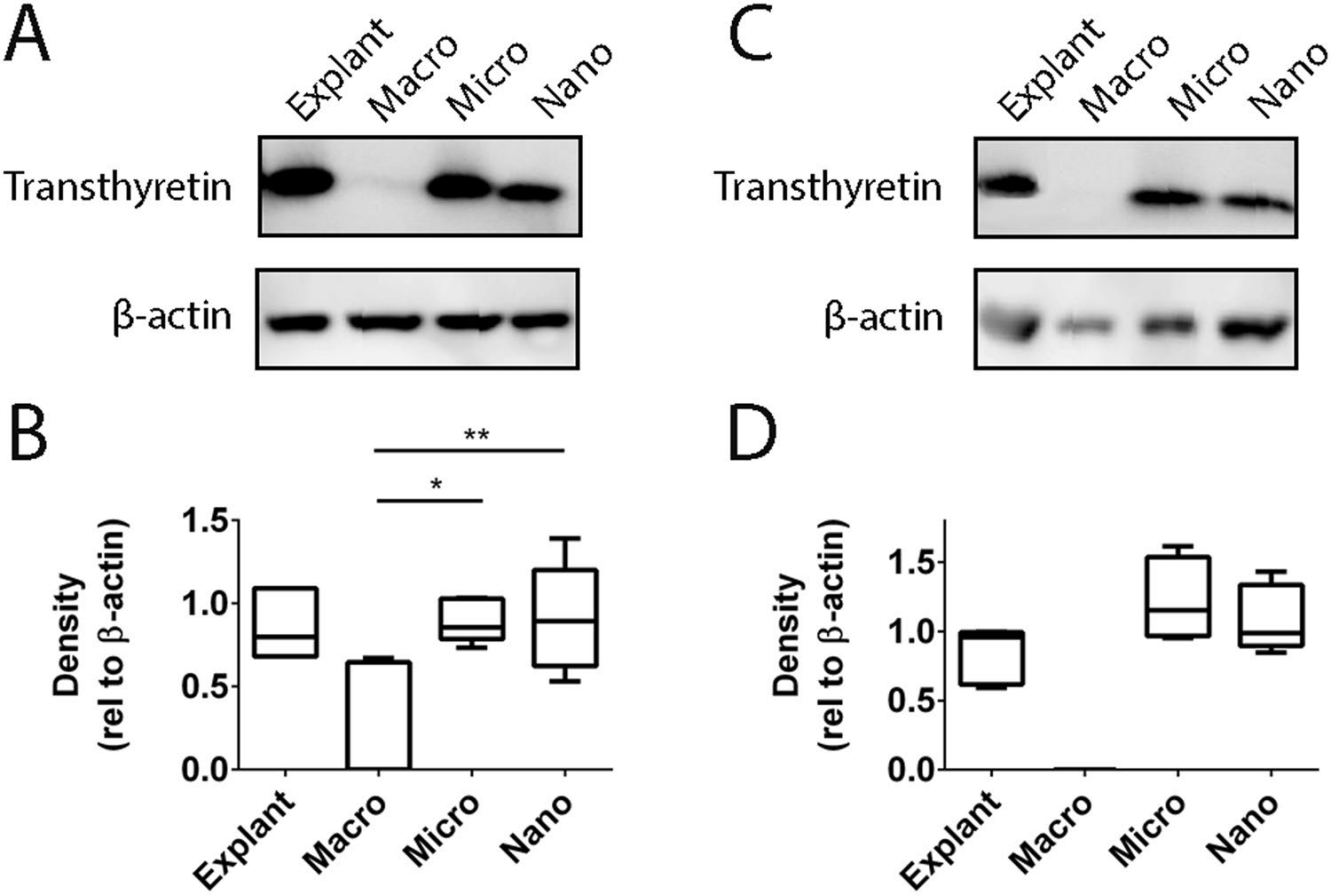
Transthyretin is carried by placental extracellular vesicles. Representative Western blots showing the presence of transthyretin in different fractions of extracellular vesicles extruded from first trimester and term placental explants (**A**,**C**). Full-length blots are presented in Supplementary Figure 2. Semi-quantitative analyses of the levels of transthyretin relative to the levels of β-actin show that there were significantly higher levels of transthyretin in micro- (*p = 0.011, n = 5) and nano- (**p = 0.0099, n = 5) vesicles compared with macro-vesicles from first trimester placentae (**B**). Transthyretin was not carried by macro-vesicles from term placentae (**D**). Data is plotted as box-whisker plots showing the median, interquartile range (box) and maximum/minimum (whiskers).

In order to determine whether extracellular vesicles from first trimester and term human placentae carry transthyretin as part of their protein cargo, macro-, micro- and nano- vesicles were collected from the same placentae by differential centrifugation, and probed for transthyretin by western blot under reducing conditions. All three types of extracellular vesicles from first trimester placentae carried transthyretin. However, when normalised to β-actin, the levels of transthyretin in macro-vesicles were significantly lower than that in micro- (p = 0.011) and nano- vesicles (p = 0.0099, n = 5, Fig. 2A,B). In contrast, transthyretin was present only in micro- and nano- vesicles extruded from term placentae but absent from macro-vesicles from the same placentae (n = 5, Fig. 2C,D). Transthyretin was also present in the first trimester and term human placental explants from which the extracellular vesicles were derived (n = 5, Fig. 2A,C).

### Extracellular vesicles from preeclamptic placentae carry altered levels of total aggregated protein

**Figure 3:**
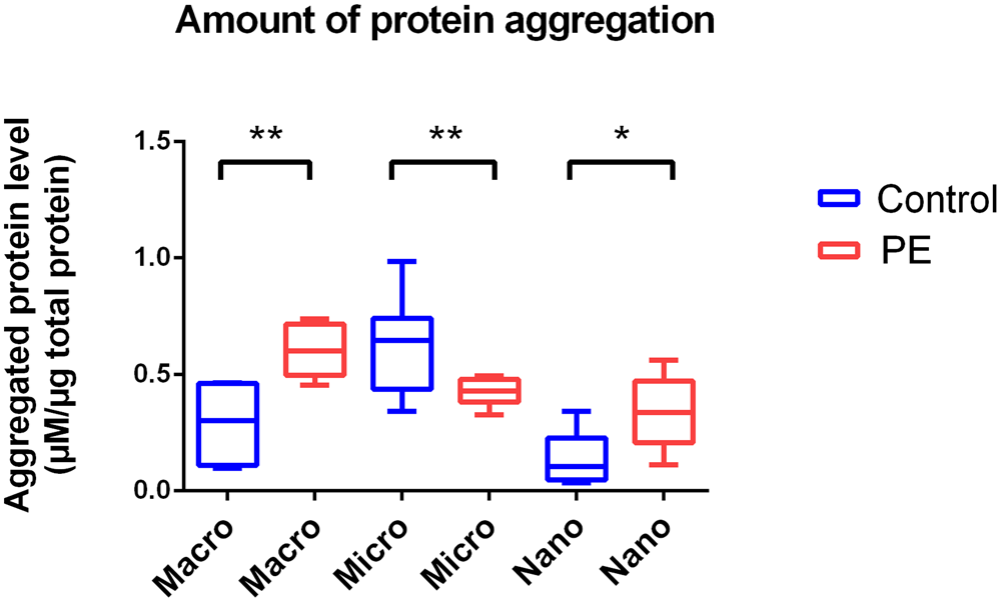
Levels of total aggregated protein in placental extracellular vesicles determined by ProteoStat Assay. The levels of total aggregated protein in macro-, micro- and nano- vesicles extruded from control and preeclamptic placentae were measured by a fluorometric ProteoStat® assay. Having normalised to the amount of protein loaded, unpaired t-tests were performed to investigate statistical differences between the levels of aggregated protein present in each fraction of extracellular vesicles from control and preeclamptic placentae (**p < 0.001, *p < 0.05). Data is plotted as box-whisker plots showing the median, interquartile range (box) and maximum/minimum (whiskers).

Since we have shown that preeclamptic placentae exhibit increased levels of aggregated transthyretin compared to normotensive placentae and aggregated proteins have been implicated in the pathogenesis of several diseases, we examined whether the levels of total aggregated proteins carried by macro-, micro- and nano- vesicles from preeclamptic placentae were increased compared to those from normotensive control placentae using the ProteoStat® Protein Aggregation Assay. In one microgram of total protein, the levels of total aggregated protein were significantly increased in macro- (0.60 ± 0.11 µM compared to 0.29 ± 0.20 µM, p = 0.0052) and nano- (0.34 ± 0.14 µM vs 0.13 ± 0.12 µM, p = 0.0114) vesicles derived from preeclamptic placentae compared with that from control placentae (mean ± SD, n = 8, Fig. 3). In contrast, the total level of aggregated protein was significantly lower in micro-vesicles derived from preeclamptic placentae compared with that from control placentae (0.42 ± 0.06 µM/µg of total protein compared with 0.63 ± 0.21 µM/µg, p = 0.0014, n = 8, Fig. 3). It is interesting that we detected increased levels of total aggregated proteins in preeclamptic macro-vesicles even though transthyretin was not significantly detected (Fig. 2), suggesting that these vesicles contained other non-transthyretin protein aggregates.

### The levels of aggregated and monomeric transthyretin were increased in nano-vesicles, but not micro-vesicles, from preeclamptic placentae compared to normotensive placentae

**Figure 4:**
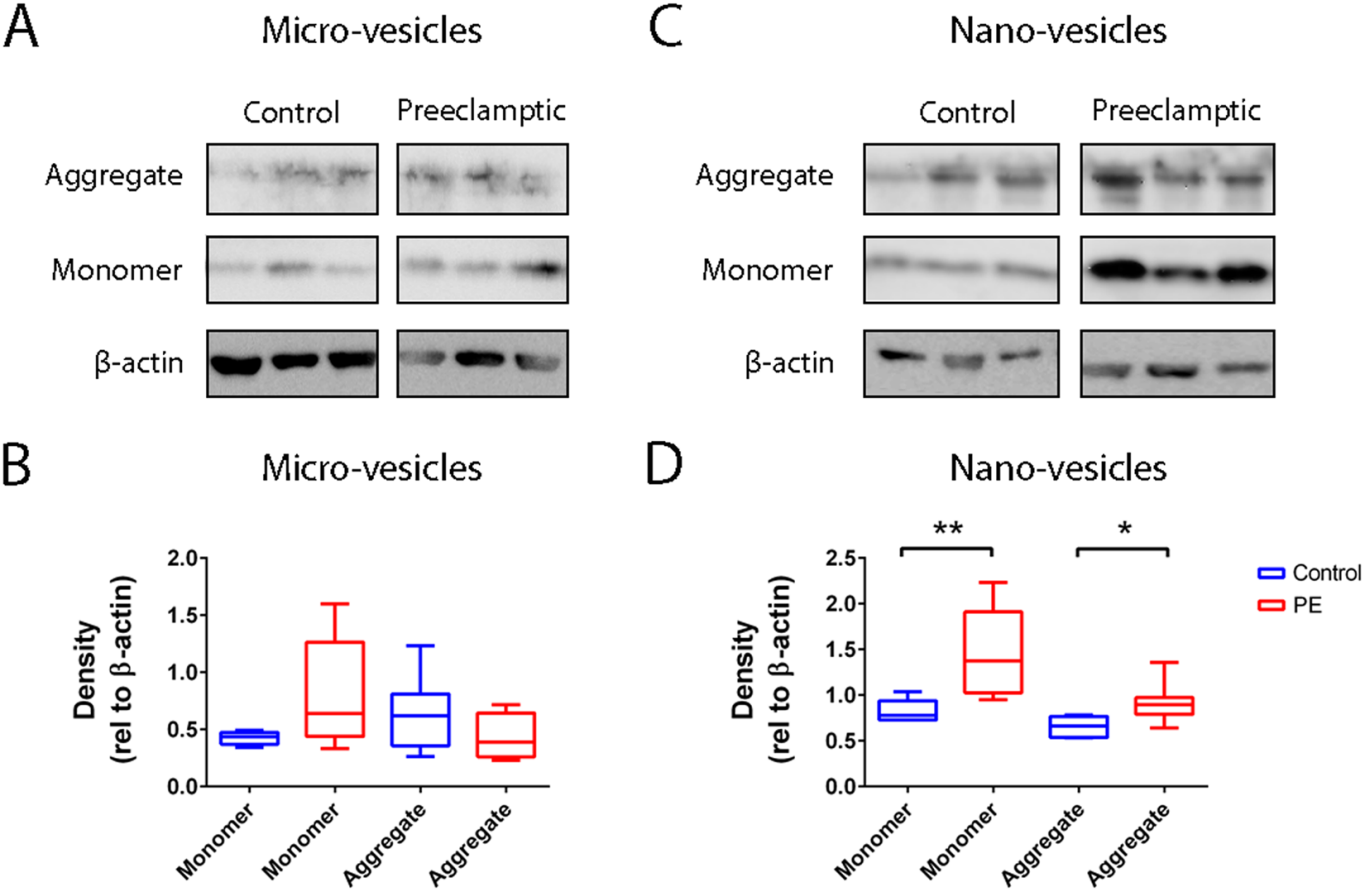
Transthyretin levels in micro- and nano- vesicles derived from normotensive and preeclamptic placentae. Western blotting of transthyretin in micro- (**A**) and nano- (**C**) vesicles from normotensive control and preeclamptic placentae was performed under non-reducing conditions. The levels of aggregated (>150 kDa) and monomeric (14 kDa) transthyretin were semi-quantitated relative to that of β-actin (**p = 0.0097, *p = 0.0176, n = 8, **B**,**D**). Full-length blots are presented in Supplementary Figure 3. Data is plotted as box-whisker plots showing the median, interquartile range (box) and maximum/minimum (whiskers).

In order to determine whether aggregated transthyretin contributed to the altered levels of total aggregated protein in extracellular vesicles from preeclamptic placentae, western blotting was performed under non-reducing conditions for transthyretin in placental extracellular vesicles. Since macro-vesicles from later gestation human placentae contain little or no transthyretin (Fig. 2C), our analysis was confined to placental micro- and nano-vesicles. Both micro- and nano- vesicles from preeclamptic and control normotensive placentae carried monomeric (14 kDa) as well as aggregated ( > 150 kDa) transthyretin. Semi-quantification by western blot showed that the levels of transthyretin were not significantly different in micro-vesicles from preeclamptic and normotensive placentae (p > 0.05, n = 8, Fig. 4A,B). In contrast, the levels of both monomeric and aggregated transthyretin were significantly increased in nano-vesicles from preeclamptic placentae compared to nano-vesicles from normotensive control placentae (p = 0.0097 and p = 0.0176 respectively, n = 8, Fig. 4C,D).

### A large proportion of transthyretin secreted by preeclamptic placentae was vesicle-associated

**Figure 5:**
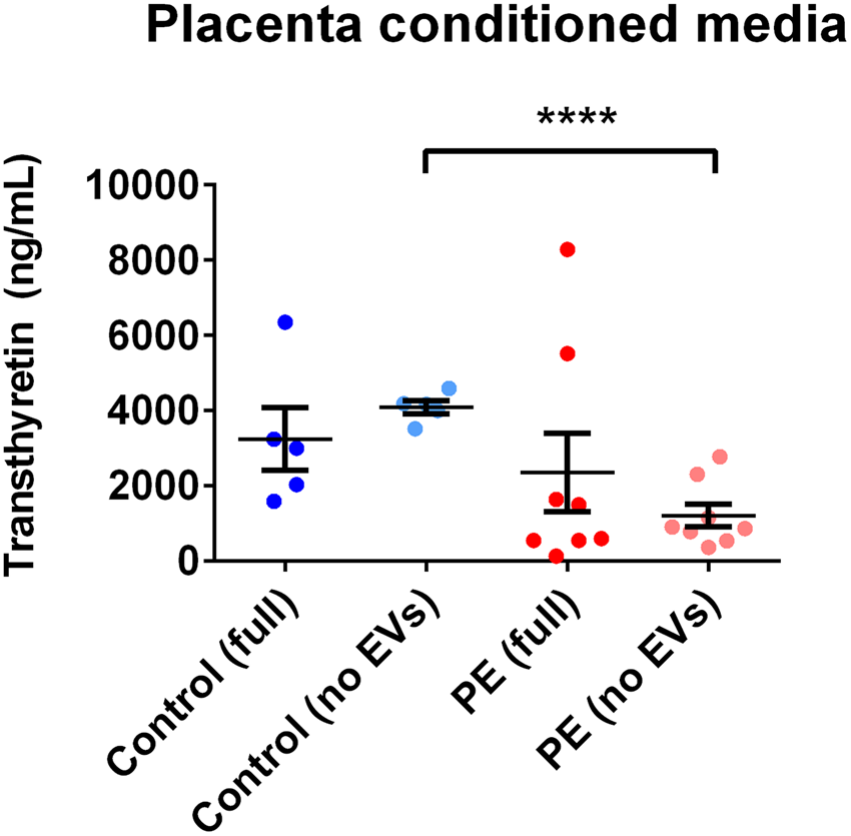
Transthyretin levels in vesicle-depleted and vesicle-replete conditioned media from preeclamptic and normotensive placentae. The concentrations of transthyretin in the conditioned media from normotensive control and preeclamptic placentae were measured by sandwich ELISA. For each sample, extracellular vesicles were depleted from half of the conditioned media by centrifugation at 100,000 g for one hour, and the vesicle-depleted (no EVs) and vesicle-replete (full) conditioned media were assayed together (****p < 0.0001). Mean and SEM are depicted.

In order to investigate how much of the transthyretin released by human placentae is associated with extracellular vesicles, conditioned media were collected from preeclamptic and normotensive control placental explants. The conditioned media were divided into two portions, one of which was ultracentrifuged at 100,000 g for one hour to deplete extracellular vesicles before assaying for transthyretin by ELISA, while the second portion was assayed directly for transthyretin. Transthyretin concentrations in the conditioned media that had not been ultracentrifuged from control and preeclamptic placentae were not significantly different (3250 ± 1864 ng/mL and 2352 ± 2949 ng/mL, respectively, mean ± SD, p > 0.05, n = 8, Fig. 5). However, depletion of extracellular vesicles from the media significantly reduced the level of transthyretin by 48% in preeclamptic (1216 ± 862 ng/mL) but not control (4094 ± 386 ng/mL) media (p < 0.0001, Fig. 5).

## Discussion

Transthyretin, a circulating transporter protein for thyroxine and retinol binding protein, has recently been implicated in the pathogenesis of preeclampsia^17, 18^. Here, we reported that transthyretin is found at increased levels in preeclamptic placentae compared to normotensive placentae, and that in preeclampsia, this transthyretin is aggregated and almost half of all transthyretin secreted by the placenta is associated with extracellular vesicles. In contrast, the mRNA levels of transthyretin in preeclamptic placentae were not altered, suggesting changes in the posttranscriptional processing of transthyretin, leading to its aggregation and packaging into placental nano-vesicles in preeclampsia.

Firstly, we confirmed the observations of previous studies showing that transthyretin can be found at increased levels in preeclamptic placentae compared to normotensive placentae^17, 19, 20^. It has been previously shown *in vitro* that the synthesis of transthyretin by trophoblasts is increased in hypoxia^21, 22^ and it is interesting to speculate that the increased levels of transthyretin observed in preeclamptic placentae may be a result of hypoxia-reperfusion injury that has been suggested to occur in preeclamptic pregnancies. However, surprisingly, while the levels of transthyretin protein were increased, the levels of mRNA encoding transthyretin remained unchanged between preeclamptic and normotensive placentae. This suggests that it is the posttranscriptional processing of transthyretin which is altered in preeclampsia, resulting in the increased protein levels observed. This hypothesis is supported by the finding of increased aggregated transthyretin in preeclamptic placentae compared to control placentae. At this stage, it remains unclear what causes transthyretin to become aggregated in preeclamptic placentae but this could potentially be due to protein misfolding. Indeed, increased levels of misfolded/amyloid protein have been detected in the urine of women with preeclampsia^23^ and we also report increased levels of total aggregated proteins in macro- and nano- vesicles extruded by preeclamptic placentae.

In this work, the granular staining pattern of transthyretin in the syncytiotrophoblast of both preeclamptic and normotensive control placentae led us to speculate whether the transthyretin produced by the human placenta may be localised to intracellular structures within the syncytiotrophoblast, such as endosomes. As extracellular vesicles are known to be produced by the syncytiotrophoblast throughout gestation and some subtypes of extracellular vesicles, such as exosomes (one type of nano-vesicles), are produced specifically through the endosomal pathway^24, 25^, we next investigated whether transthyretin may be part of the protein cargo of placental extracellular vesicles. Indeed, in a previous proteomic screen of macro-, micro- and nano- vesicles from first trimester human placentae, transthyretin was identified in each of the three classes of placental extracellular vesicles^26^. Here we corroborate that report, further showing that while all three size fractions of extracellular vesicles from first trimester human placentae carry transthyretin, levels were significantly higher in micro- and nano- vesicles compared with macro-vesicles. Furthermore, only micro- and nano- vesicles from term placentae carried readily detectible levels of transthyretin. The differential levels of transthyretin in the three vesicle types suggests the process of incorporation of transthyretin into placental extracellular vesicles is not random but rather that there is specific packaging of transthyretin into the smaller vesicles.

In non-pregnant individuals, transthyretin is mainly secreted by the liver, choroid plexus, pancreas and retina^27^, and it plays an important role in transporting thyroxine and retinol. Thyroxine is the inactive form of thyroid hormone that can be converted to active triiodothyronine which plays crucial roles in controlling metabolism, appetite and digestion, and brain development. In the first trimester of pregnancy, the human fetus is unable to synthesise its own thyroid hormone, therefore a large supply of thyroxine is required from the mother and placenta for fetal growth and brain development^14, 28^. The human placenta secretes transthyretin throughout gestation^12, 13, 19, 28^ and it has been proposed that a transthyretin shuttle system exists to deliver maternal thyroid hormone through the placenta to the fetus^11, 22, 28^. How vesicle-associated transthyretin may play into such a shuttle system requires further investigation, however it is possible that these transthyretin-rich placental extracellular vesicles represent a circulating scaffold on which thyroxine and retinol can bind, creating an alternative pathway for the transport of maternal thyroxine to the fetus. Interestingly, human trophoblasts have been shown to be able to take up placental extracellular vesicles *in vitro* ^29^.

Here, we showed that both macro- and nano- vesicles produced by preeclamptic placentae carried more total aggregated protein compared to control placentae, and for placental nano-vesicles, one of these aggregated proteins was transthyretin. Since increased levels of transthyretin were only detected in nano-vesicles, and not larger vesicles, from preeclamptic placentae, this again suggests specific targeting of excess/misfolded/aggregated transthyretin into this vesicle type, further emphasizing the different intracellular pathways that are utilised by the syncytiotrophoblast to produce different types of extracellular vesicles. The isolation methodology that we used in this study produces a nano-vesicle fraction consisting of both exosomes, which are produced via the endosomal pathway, as well as other small membranous vesicles, which likely bleb from the syncytiotrophoblast apical membrane. We cannot distinguish whether the increased levels of aggregated transthyretin were carried in exosomes or the other nano-vesicles^30^.

Not only are different vesicle types produced via different mechanisms but they also carry different eat-me and don’t eat-me signals that may allow them to interact with different recipient cell types with different kinetics^26^. Indeed, we have recently shown that micro- and nano-vesicles target to different maternal organs *in vivo* ^31, 32^. As aggregated proteins are^33^ cytotoxic and can affect trophoblast function^17, 33^, the presence of aggregated transthyretin in placental nano-vesicles has at least two major implications. First, packaging of aggregated transthyretin into nano-vesicles may allow the syncytiotrophoblast to rid itself of potentially damaging misfolded/aggregated proteins which may contribute to endoplasmic reticulum (ER) stress, leading to the production of more misfolded proteins^34^. In this sense, the packaging of aggregated proteins into nano-vesicles may be a mechanism to dispose of toxic aggregated proteins in order to protect the syncytiotrophoblast. But perhaps more importantly, in light of the finding that placental nano-vesicles localise to specific maternal organs *in vivo*, the presence of aggregated transthyretin in these vesicles may allow targeted delivery of these toxic proteins to specific maternal organs, conducting a signal of cellular stress from the placenta to particular maternal organs. If aggregated transthyretin can indeed induce ER stress in recipient cells and preeclamptic placental nano-vesicles are targeting these messages to specific organs and cell types, then this protein carried by placental extracellular vesicles may very well be an important toxin produced by the placenta that contributes to the pathogenesis of preeclampsia. This suggestion is supported by reports that aggregated proteins are pathogenic in several other diseases^35–37^.

In summary, transthyretin is an important protein for healthy fetal growth and development. In preeclampsia, the placental production of this protein is affected at the post-transcriptional level and this in turn leads to increased packaging of aggregated transthyretin into placental nano-vesicles. Whether the packaging of aggregated transthyretin, and other protein aggregates, into placental nano-vesicles has functional consequences in the pathogenesis of preeclampsia remains to be determined.

## Materials and Methods

### Patient sample collection

This study was approved by the Auckland Regional Health and Disabilities Ethics Committee and all methods were performed in accordance with the relevant guidelines and regulations. Placentae and blood plasma samples were obtained from Greenlane Hospital (Auckland, NZ) and Auckland City Hospital (Auckland, NZ) with informed written consent.

First trimester placentae between 8–12 weeks of gestation were collected after elective surgical termination of pregnancy for socio-psychological reasons. Preeclamptic and gestation-matched normotensive control placentae were collected within two hours of delivery. Preeclampsia was defined following the guidelines of the American College of Obstetricians and Gynaecologists as new-onset hypertension (maternal systolic blood pressure ≥140 mmHg and/or diastolic blood pressure ≥90 mmHg on two occasions separated by six hours) and proteinuria (>300 mg of protein in a 24-hour urine collection period) after 20 weeks of gestation^1^.

All preeclamptic placentae used for this study were from patients with severe preeclampsia (systolic and/or diastolic blood pressures of ≥160 mmHg and 110 mmHg, respectively): five had early-onset preeclampsia (symptoms arising ≤32 weeks of gestation) and three had late-onset preeclampsia. Of the eight preeclamptic patients, seven delivered FGR infants. The patients’ clinical characteristics are summarised in Table 1.

**Table 1:** Clinical characteristics of recruited patients.

	Preeclamptic (n = 8)	Control (n = 8)	p value
Maternal age (years and range)	36.1 (32–40)	35.3 (28–44)	p > 0.05
Gestation weeks at delivery	31^+3^ (24–37^+2^)	37^+5^ (36–39^+6^)	p < 0.05
Systolic BP (mmHg)	161 (150–170)	<140	p < 0.05
Diastolic BP (mmHg)	104 (90–118)	<90	p < 0.05
Proteinuria	>+++	—	p < 0.05
IUGR	7	0	p < 0.05

### Collection of placental extracellular vesicles

Placental extracellular vesicles were collected from first trimester, preeclamptic and term placentae following previously published methods^26, 38^. Briefly, four placental explants of around 400 mg were dissected from each placenta and cultured in Netwell^TM^ inserts (Corning, NZ) in Advanced DMEM/F12 medium supplemented with 2% FBS and 1% Penicillin/Streptomycin (Invitrogen, NZ) in ambient oxygen with 5% CO_2_ at 37 °C.

After 16 hours, the culture medium was aspirated from the culture wells and placental extracellular vesicles were collected by differential centrifugation (Avanti J30I Ultracentrifuge, JA 30.50 Ti fixed angle rotor, Beckman Coulter, NZ). The culture medium was sequentially centrifuged at 2,000 g for five minutes to collect macro-vesicles, 20,000 g for one hour to collect micro-vesicles, and 100,000 g for one hour to collect nano-vesicles. Contaminating red blood cells were removed from the macro-vesicle fraction by hypotonic lysis in ultrapure water (EMD Millipore, NZ) and contaminating leukocytes were removed using anti-CD45 magnetic beads (Invitrogen, NZ).

### Protein extraction from placental explants and extracellular vesicles

Total protein was extracted from placental explants by homogenising a 0.5 cm^3^ block of villous placental tissue in one millilitre of RIPA buffer (50 mM Tris, 150 mM NaCl, 1% sodium deoxycholate, 0.1% SDS, 1% Nonidet P40 substitute, 1 mM PMSF, pH 7.4) supplemented with protease inhibitor (Roche, USA) using a rotor-stator homogeniser (John Morris Scientific, NZ). The homogenate was centrifuged at 13,000 g for ten minutes at 4 °C to remove unlysed tissue and membranes, and the supernatant (protein lysate) was stored at −80 °C until use.

Total protein was extracted from each fraction of placental extracellular vesicles by manual pipetting using RIPA buffer supplemented with protease inhibitor and stored at −80 °C until use. The protein content of each lysate was quantified by the Pierce^TM^ bicinchoninic acid protein assay (Thermo Fisher Scientific, NZ).

### Detection of total aggregated protein levels

The amount of total aggregated protein in macro-, micro- and nano- vesicles was measured by the ProteoStat® Protein Aggregation Assay (Enzo Lifesciences, NZ) following previously described methods^39^. Briefly, ten micrograms of protein lysates were loaded in duplicate in black-bottom 96 well microplates and the ProteoStat® detection dye was added. Samples were incubated in the dark for 15 minutes and fluorescence was measured at 485/620 nm. A sample of aggregated lysozyme and native lysozyme were included in the assay as a positive and negative control, respectively.

### Western blotting

Total protein from placental explants and extracellular vesicles were resolved on 14% polyacrylamide SDS-PAGE gels under reducing or non-reducing conditions. Protein lysates were transferred to Hybond^TM^-C extra nitrocellulose membranes (Amersham Biosciences, UK). Successful protein transfer was confirmed by staining with 0.1% Ponceau S (w/v). Membranes were blocked with 5% non-fat milk powder (w/v) before incubating with a rabbit polyclonal antibody against human transthyretin (1:500, DAKO, US), rabbit serum IgG as a control, or a mouse monoclonal antibody against β-actin (1:4000, Abcam, NZ). Membranes were then incubated with the corresponding HRP-conjugated anti-rabbit or anti-mouse antibody (Jackson ImmunoResearch, USA) and the presence of target proteins were detected using Amersham^TM^ ECL^TM^ Prime detection reagent and visualised on Image Quant LAS3000 (GE Healthcare, UK). Images were annotated using Adobe® Photoshop® Elements 5.0. Protein abundance was semi-quantified by densitometry relative to β-actin using the Kodac Digital Science 1D image analyser (Kodac, Japan).

### Immunohistochemistry

Formalin-fixed placental tissue from normal or preeclamptic pregnancies were sectioned at 10 μm and deparaffinized with Citrisolv (Fisher Scientific, USA). Antigen retrieval was performed by heating the sections in 0.1 M sodium citrate (pH 6.0). Sections were blocked in blocking buffer (3% bovine serum albumin and 0.1% Triton X-100 in PBS) and then incubated with transthyretin polyclonal rabbit antibody (DAKO) overnight at 4 °C. Primary antibody-bound target proteins were visualized with goat anti-rabbit Alexa-Fluor 488 (Invitrogen). The specificity of transthyretin antibody was confirmed by blocking transthyretin staining by incubating the primary antibody with the immunogenic peptide or by using normal rabbit serum IgG instead of primary antibody. Images were processed with brightness/contrast adjustment using Photoshop CS2 (Adobe) at the same levels.

### Quantitative RT-PCR

Placental tissue was homogenised in TRIzol® and RNA was extracted using the Purelink® RNA Mini kit (Invitrogen, NZ). RNA was eluted in DEPC-treated water and the amount of RNA present was quantified spectrophotometrically at 260 nm using a Nanodrop 2000 Spectrophotometer (Thermo Fisher Scientific, NZ).

One microgram of RNA was used for cDNA synthesis. Potential genomic DNA contamination was first removed by treatment with DNase I (Sigma-Aldrich, NZ) and synthesis of cDNA was then performed using the SuperScript^TM^ III First-Strand Synthesis System (Invitrogen, NZ). The resultant cDNA was stored at −20 °C.

Real-time PCR primers for human *TTR, UBC, ACTB and RPLP0* were designed using Primer3^40^. The primer sequences and amplicon sizes are summarised in Table 2. Quantitative RT-PCR was carried out in the QuantStudio^TM^ 12 K Flex Real-time PCR machine (Applied Biosystems, USA) using 10 µL reaction volumes consisting of 5 μL Platinum® SYBR® Green qPCR SuperMix-UDG w/ROX (Invitrogen, NZ), 1 μL of each primer working solution (5 μM), 1 μL water and 2 μL cDNA. Each sample was run in triplicate and threshold cycle values (<35 cycles) that differed by less than 0.5 cycles were averaged for analysis. Raw measurements were normalised to the geometric mean of three reference genes, *UBC, ACTB and RPLP0*, and the fold change of *TTR* mRNA between control and preeclamptic placentae was calculated accordingly.

**Table 2:** Sequences of primers used for qRT-PCR.

Gene of interest	Forward primer (5′–3′)	Reverse primer (5′–3′)	Product (bp)
*TTR*	GCATGCAGAGGTGGTATTCA	GTCCCTCATTCCTTGGGATT	123
*UBC*	GGGCACTGGTTTTCTTTCCA	AGAATCGCCGACAAGGGACTA	65
*ACTB*	GCGGACTATGACTTAGTTGCGTTA	CATCTTGTTTTCTGCGCAAGTT	67
*RPLP0*	ATGGGCAAGAACACCATGATG	CCTCCTTGGTGAACACAAAGC	118

### ELISA

One placental explant of approximately 400 mg was prepared from each preeclamptic and control placenta and cultured for 16 hours at 37 °C in 95% air/5% CO_2_. After thoroughly mixing, one millilitre of the conditioned media was harvested from each sample and divided into two portions. One portion was centrifuged at 100,000 g for one hour at 4 °C before storage at −80 °C while the other portion was directly stored at −80 °C without further processing.

The human PreAlbumin/Transthyretin ELISA kit (ab108895, Abcam, NZ) was used to measure the amount of transthyretin in the conditioned medium. This kit has a limit of detection of 0.03 ng/mL and an intra- and inter- assay coefficient of 4.9% and 7.4%, respectively. To be within the dynamic range for detection, all placental conditioned media samples were diluted 1:30 prior to quantification.

### Statistical analysis

As all data was shown to be normally distributed by the Kolmogorov-Smirnov and Shapiro Wilk normality tests, paired and unpaired t-tests were performed to assess statistical significance on GraphPad PRISM 6.01 as appropriate (GraphPad Software Inc., USA). A p value < 0.05 was considered statistically significant. Data was plotted as box-whisker plots showing the maximum/minimun, interquartile range and median.

## Electronic supplementary material

Full Western blots
