# Supplementary material for: Aggregated transthyretin is specifically packaged into placental nano-vesicles in preeclampsia
Source: Sci Rep. 2017 Jul 27;7:6694. doi: 10.1038/s41598-017-07017-x (PMC5532246; doi:10.1038/s41598-017-07017-x)
Supplement: Supplementary file 1 — Full Western blots [file 41598_2017_7017_MOESM1_ESM.doc]

**Aggregated transthyretin is packaged specifically into placental nano-vesicles in preeclampsia (Supplementary Figures)**

Mancy Tong^1,*^, Shi-bin Cheng^2^, Qi Chen^1^, Joana DeSousa^3^, Peter R. Stone^1,3^, Jo L. James^1^, Larry W. Chamley^1^ & Surendra Sharma^2^

^1^ Department of Obstetrics and Gynaecology, The University of Auckland, Auckland, 1142, New Zealand.

^2^ Department of Pediatrics, Women and Infants Hospital-Warren Alpert Medical School of Brown University, Providence, Rhode Island, USA

^3^ Maternal Fetal Medicine, Auckland City Hospital, New Zealand

**Supplementary Figure 1: Transthyretin expression by preeclamptic placentae**

Representative full Western blots showing the levels of transthyretin present in three control and preeclamptic placental lysates.


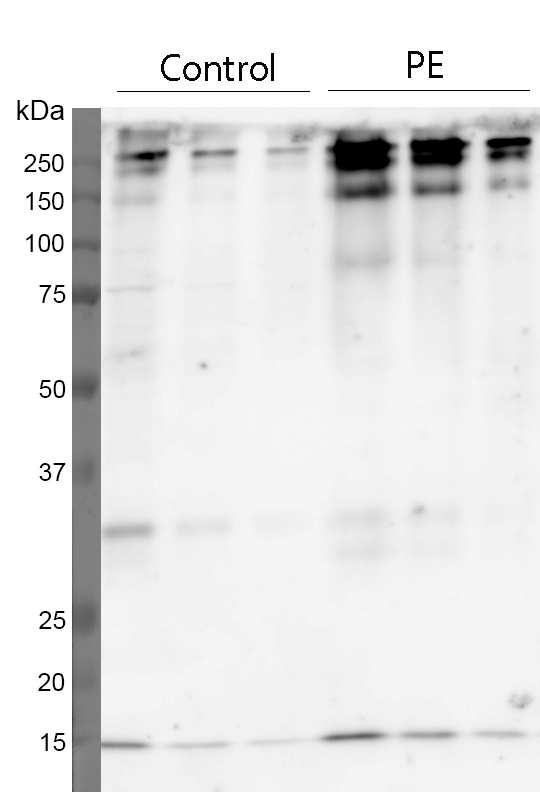


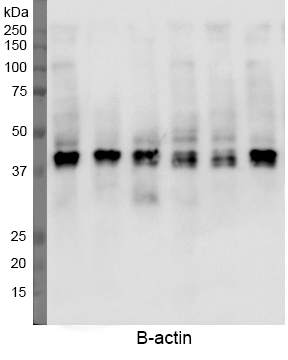


**Supplementary Figure 2: Transthyretin is carried by placental extracellular vesicles**

Representative full Western blots showing the presence of transthyretin in micro- and nano- vesicles from first trimester (A) and term (B) human placentae.

**
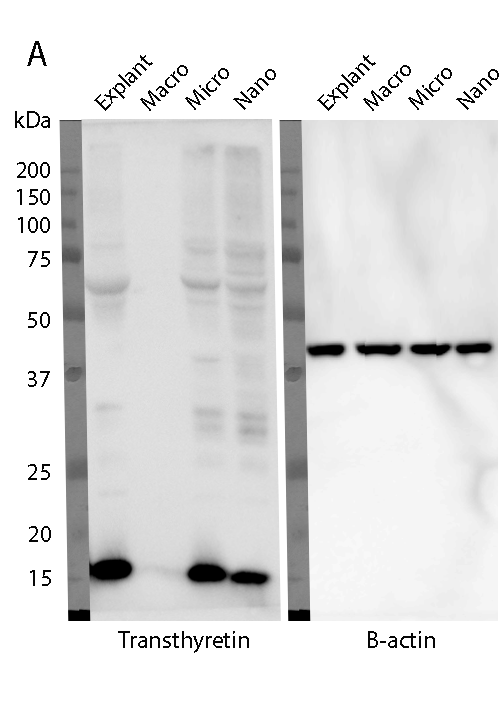

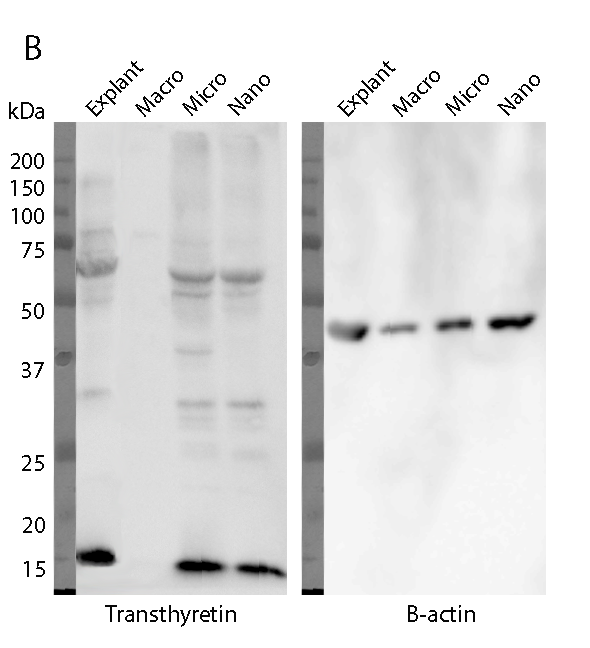
**

**Supplementary Figure 3: Transthyretin levels in micro- and nano- vesicles derived from normotensive and preeclamptic placentae**


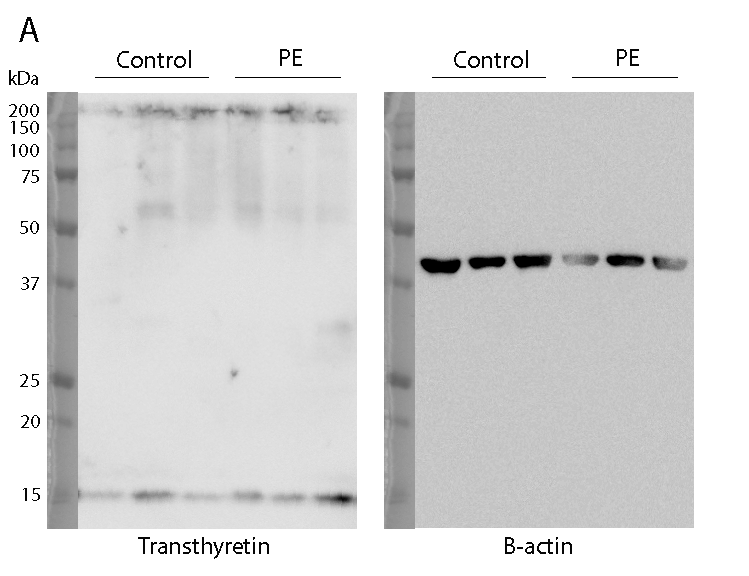

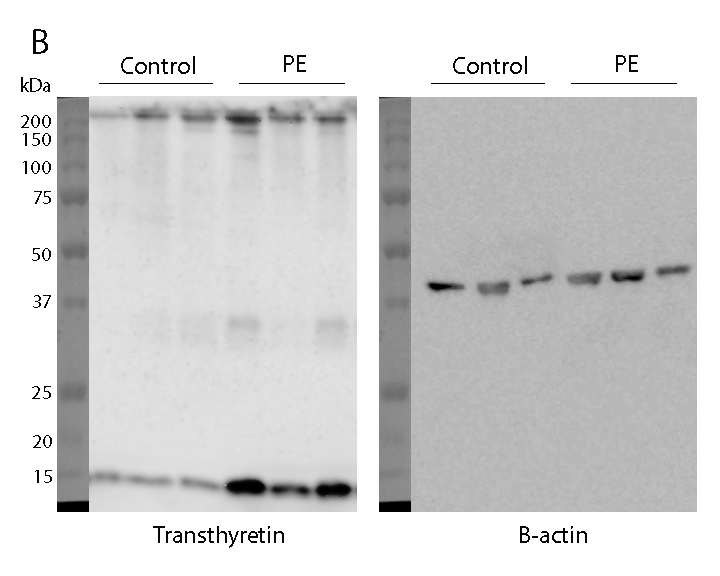
Representative full Western blots showing the presence of aggregated and monomeric transthyretin in micro- (A) and nano- (B) vesicles derived from three control and preeclamptic placentae.
